# Qualitative analysis of patient-centered decision attributes associated with initiating hepatitis C treatment

**DOI:** 10.1186/s12876-015-0356-5

**Published:** 2015-10-01

**Authors:** Jessica L. Zuchowski, Alison B. Hamilton, Jeffrey M. Pyne, Jack A. Clark, Aanand D. Naik, Donna L. Smith, Fasiha Kanwal

**Affiliations:** 1 VA HSR&D Center for the Study of Healthcare Innovation, Implementation & Policy, 16111 Plummer St. Bldg. 25, North Hills, CA 91343 USA; 2 Department of Psychiatry and Biobehavioral Sciences, David Geffen School of Medicine at UCLA, Los Angeles, CA 90095 USA; 3 Center for Mental Healthcare and Outcomes Research, Central Arkansas Veterans Healthcare System, 2200 Fort Roots Drive, North Little Rock, AR 72114 USA; 4 Psychiatric Research Institute, University of Arkansas for Medical Sciences, 4300 West 7th Street, Little Rock, AR 72205 USA; 5 Center for Healthcare Organization and Implementation Research, Edith Nourse Rogers Memorial Veterans Hospital, 200 Springs Rd, Bedford, MA 01730 USA; 6 Department of Health Policy and Management, Boston University School of Public Health, 715 Albany St. #358w, Boston, MA 02118 USA; 7 VA HSR&D Center for Innovations in Quality, Effectiveness & Safety, Michael E DeBakey VA Medical Center, 2002 Holcombe Boulevard, Houston, TX 77030 USA; 8 Department of Medicine, Baylor College of Medicine, One Baylor Plaza, Houston, TX 77030 USA

**Keywords:** Chronic hepatitis C viral infection (CHC), Anti-viral treatment, Decision attributes, Shared decision-making aid, Veterans

## Abstract

**Background:**

In this era of a constantly changing landscape of antiviral treatment options for chronic viral hepatitis C (CHC), shared clinical decision-making addresses the need to engage patients in complex treatment decisions. However, little is known about the decision attributes that CHC patients consider when making treatment decisions. We identify key patient-centered decision attributes, and explore relationships among these attributes, to help inform the development of a future CHC shared decision-making aid.

**Methods:**

Semi-structured qualitative interviews with CHC patients at four Veterans Health Administration (VHA) hospitals, in three comparison groups: contemplating CHC treatment at the time of data collection (Group 1), recently declined CHC treatment (Group 2), or recently started CHC treatment (Group 3). Participant descriptions of decision attributes were analyzed for the entire sample as well as by patient group and by gender.

**Results:**

Twenty-nine Veteran patients participated (21 males, eight females): 12 were contemplating treatment, nine had recently declined treatment, and eight had recently started treatment. Patients on average described eight (range 5–13) decision attributes. The attributes most frequently reported overall were: physical side effects (83 %); treatment efficacy (79 %), new treatment drugs in development (55 %); psychological side effects (55 %); and condition of the liver (52 %), with some variation based on group and gender. Personal life circumstance attributes (such as availability of family support and the burden of financial responsibilities) influencing treatment decisions were also noted by all participants. Multiple decision attributes were interrelated in highly complex ways.

**Conclusions:**

Participants considered numerous attributes in their CHC treatment decisions. A better understanding of these attributes that influence patient decision-making is crucial in order to inform patient-centered clinical approaches to care (such as shared decision-making augmented with relevant decision-making aids) that respond to patients’ needs, preferences, and circumstances.

## Background

Chronic infection with hepatitis C virus (CHC) is a large public health problem, impacting an estimated three million people in the United States [1, 2]. Successful treatment slows disease progression and reduces the risk of cirrhosis and liver cancer, which often are fatal [3–10]. Historically, antiviral treatment was lengthy and associated with substantial side effects, with only a modest chance of success. However, CHC medications are rapidly changing with the introduction of new direct acting antiviral agents which can be combined with older therapies to reduce use of interferon, or replace interferon therapy entirely. With these new treatment options, treatment duration is attenuating for most patients, with associated side effects becoming less prevalent and less severe [11–13]. However, the new medications are also substantially more expensive [14]. In addition, with many drugs in the development pipeline, future treatments are anticipated to continue to improve and offer new and potentially better choices [15].

Providers and patients face a new context for CHC treatment decision-making. Previously the decision to treat or delay treatment was framed by tradeoffs among the severity of side effects, treatment duration, and efficacy [16–18]. Now patients and providers must consider a rapidly changing set of medication options, a constant horizon of potentially better medications, and extremely costly medications [19]. Given these considerations, watchful waiting may be appropriate for patients with early stage disease (0/1 stage fibrosis) and significant comorbidity. As a result, decision-making around CHC treatment remains complex and many patients are offered deferral of treatment as an appropriate option.

Patient-centered care postulates that patients’ understanding of outcomes expectancies, potential risks and benefits, and values-informed preferences should guide treatment decisions, in addition to the clinician’s expert opinion and evidence [20, 21]. Data suggest that health outcomes are comparatively better for patients who are informed about their treatment options, have realistic expectations of outcomes, participate in setting goals for potential treatments, and are able to link personal values to their goals [22, 23]. Shared decision-making is a model of patient-centered care that enables and encourages patients to play an active role in the management of their own health [24, 25]. Well-developed, high-quality, formal decision aids can augment the process of shared decision-making and enhance patient involvement in the decision-making process during clinical encounters [26].

Decision-making aids actively involve the patient in the shared decision-making process and thus, must be based not only on accurate scientific information, but also speak to patient perceptions which may influence the treatment decision. To inform the development of a relevant CHC treatment decision aid, it is first necessary to identify specific decision attributes that CHC patients take into account when faced with a treatment decision. Previous work that has examined the attributes that influence patient CHC treatment decisions suggests a complex array of decision attributes, including both patients’ perceptions of medical attributes related to treatment such as treatment effects, as well as their perceptions of the impact of treatment on personal life circumstances; little work has attempted to describe how these decision attributes may be related to each other [17, 27–32].

For exploratory purposes, we used qualitative interviews with CHC patients in close proximity to their own CHC treatment decisions in order to provide a grounded description of the specific attributes that influence patients’ decisions around CHC treatment, and relationships between those attributes. For the purposes of this paper, we define a decision attribute as a patient perception of treatment, or a broader personal impact of treatment, that influences the treatment decision outcome. We identified key patient-reported attributes in treatment decision-making and explored the relationships between these attributes. We suggest that a grounded understanding of the relationships between decision attributes will be beneficial in the development of a valid and relevant decision-making aid for CHC patients.

## Methods

### Design and sample

We conducted semi-structured qualitative interviews with CHC patients at four VHA medical centers. Enrollment criteria were patients with a CHC diagnosis seeking care at one of the four VHA medical centers in the study. Exclusion criteria were 1) patients receiving CHC treatment at non-VA facilities, and 2) patients who initiated their current course of treatment more than 15 weeks prior to the interview. Patients were not excluded on the basis of previous treatment for CHC, co-infection with HIV or hepatitis B, or active or past substance or alcohol use. In order to compare men’s and women’s perspectives, we oversampled women to account for the predominance of males in VHA.

Patients were sampled from each of three stages of the decision-making process based on medical record notes entered by their CHC provider. Group 1 was contemplating CHC treatment at the time of the interview, Group 2 had recently declined treatment, and Group 3 had begun treatment within the past 15 weeks. Groups of patients and potential participants within the groups were identified by clinicians in CHC clinics and invited in person and by letter to participate. The groups facilitated comparison of decision-making processes among the groups as well as perceptions of outcomes. From a clinical standpoint, we expected that the responses from the three patient groups would provide complementary insight into the decision attributes that patients consider in making treatment decisions as well as perceptions of outcomes.

### Procedures

Interviews were conducted over the telephone. We used a semi-structured interview guide developed collaboratively by the research team (comprised of clinicians including a hepatologist, an internist, and a mental health provider, health services researchers, and social scientists) early in the project development phase, and based on the CHC treatment and patient decision-making literatures. The semi-structured nature of the guide allowed the interviewer to follow relevant topics introduced by the interviewee and open new lines of inquiry when appropriate. The interviews addressed an array of topics pertaining to patients’ perceptions of CHC and its treatment, and patients’ specific considerations when thinking about starting antiviral treatment.

We first asked patients to share their personal CHC stories: “How did you find out you had hepatitis C?” and “What does having hepatitis C mean to you?” and “How does it affect your everyday life?” Next, we asked specifically about their decision-making process around initiating treatment: “Could you describe your thoughts about treatment since the time you first found out you have it?” and “How did you come to think about starting treatment now?” Next, we asked the primary question about the most important decision attributes: “What are/were the most important considerations (or factors) for you when you think/were thinking about whether or not to start antiviral treatment?” The interviewer (JZ) used an iterative respondent debriefing process [33] in which she repeated back to the interviewee the decision attributes named, requested confirmation, and invited additional clarification and elaboration. Patients’ knowledge about CHC infection, disease progression, and available treatments was assessed indirectly through their responses to interview guide questions.

Written informed consent was obtained from all participants for participation in the study. All procedures were approved by VA Central Institutional Review Board (HH QUERI RRP: 12–194; Development of a Shared Decision-Making Aid for Hepatitis C Treatment).

### Analysis

All interviews were audio recorded and professionally transcribed. A summary template was developed based on the interview guide to capture key points in each domain of the interview guide. Four transcripts were independently summarized by five team members (JZ, AH, JC, DS, FK). Summaries were compared in a matrix [34] and discrepancies resolved by consensus. The remaining transcripts were then summarized by the lead author (JZ).

Transcripts were coded by the lead author in ATLAS.ti (Scientific Software Development, version 6) and spot-checked by a second team member. We utilized the constant comparative approach in our analysis [35] consistent with Grounded Theory [36]. In this approach, discrete narratives are compared within common themes which are integrated across narratives in order to articulate a theory of how the themes are related to each other. The analytic focus of “decision attributes” was derived *a priori* from the interview guide. We coded responses to the primary question about patients’ most important attributes and examined the entirety of each interview to identify additional attributes expressed elsewhere in participants’ accounts.

As coding progressed, coded words and phrases were compared and grouped into respective themes, and the codebook and its definitions were revised in an iterative process. Attribute themes were compiled, tallied, and ranked by frequency. Results were synthesized by comparing and contrasting coded segments of top decision attribute themes overall, by group, and by gender. Average number of decision attributes considered was calculated overall and by group. Percentage frequency of attributes was calculated and compared by group and by gender.

Using axial coding [37], relationships between the attribute themes were analyzed for co-occurrence (i.e., degree of overlap). Analysis for patterns of co-occurrence resulted in Glaser’s “theoretical properties of the category” which included distinct clusters of co-occurrence. Examples of these prominent patterns were selected for visual display in a schematic (“network diagram”) depicting interrelationships of decision attribute themes and suggesting possible hypotheses for how multiple attributes influence patients’ CHC treatment decisions.

## Results

Of the 39 patients recruited, 31 (79.5 %) agreed to participate in the study and 29 interviews were completed. The most common reason for declining to participate was inability to be reached to complete the consent process. Interviews lasted 45–60 min on average. The sample is described in Table 1. Thematic saturation was reached at 29 participants when no new decision attributes were being reported.

**Table 1:** Description of the sample (*n* = 29)

	*n*(%)
Group	
Group 1	12 (41)
Group 2	9 (31)
Group 3	8 (28)
Cirrhosis	
Group 1	6 (21)
Group 2	2 (7)
Group 3	1 (3)
Gender	
Male	21 (72)
Age	
Under 45	2 (7)
45–55	6 (21)
56–65	21 (72)
Time since CHC diagnosis	
<1 year	4 (14)
1–5 years	15 (52)
6–10 years	8 (28)
>10 years	2 (7)

### Most frequently reported decision attributes

Across the 29 patients interviewed, we identified a total of 35 decision attributes that patients considered while making treatment decisions. At the time of the interviews, all CHC treatments offered still involved interferon, though most patients were aware that new drugs were in development. On average, patients mentioned eight (range of 5 to 13) attributes. The decision attributes most frequently reported by patients were: physical side effects of antiviral treatment (*n* = 24, 83 %); treatment efficacy (*n* = 23, 79 %), new antiviral drugs in development (*n* = 16, 55 %); psychological side effects of antiviral treatment (*n* = 16, 55 %); and the condition of the liver (*n* = 15, 52 %). All patients also identified at least one attribute related to personal life circumstances, while the actual attributes named varied according to patients’ individual situations. The more common attributes which related to patient life circumstances included family and friend support network, quality of life, and personal financial pressures. The 24 most frequently reported decision attributes, which were reported by more than one patient, are listed in the saturation grid, Table 2.

**Table 2:** Saturation grid: Decision attributes considered in CHC treatment decisions (*n* = 29)

Decision Attribute	*n*	%
Physical side effects	24	83 %
Efficacy/Cure rate	23	79 %
New drugs in development	16	55 %
Psychological/Mental health side effects	16	55 %
Condition of liver	15	52 %
Treatment regimen	12	41 %
Support network/family help	12	41 %
Other illnesses	12	41 %
Quality of life	11	38 %
Length of treatment	9	31 %
Financial pressures/being able to work	7	24 %
Age/life span	7	24 %
Doctor’s recommendation	7	24 %
Medical privacy/stigma	7	24 %
Fear of transmitting illness	6	21 %
Urgency to treat	6	21 %
Trust in provider	5	17 %
Travel to VHA	5	17 %
Location/stability of living situation	5	17 %
Caregiver responsibilities	4	14 %
Quitting drinking/drugs	4	14 %
Not wanting biopsy	3	10 %
Other people’s experiences with treatment	3	10 %
Using alternate/natural treatment/herbs	3	10 %

#### Physical side effects

Patients expressed general concern that interferon-based antiviral treatment can cause severe side effects. The most common specific physical side effects mentioned were fatigue, flu-like symptoms, and nausea. Many also expressed concern around doctors’ inability to predict the severity of side effects that they might experience, if they chose to undergo treatment. There was variation in how physical side effects influenced patients’ decisions. They were an impetus behind delaying or declining treatment for some patients (e.g., “If there were some side effects to it, I’d rather not take it”), while others imagined the side effects would be manageable (e.g., “I’m still a little bit leery about the nausea and stuff but I really believe it’s manageable”), and still others saw them simply as an unavoidable aspect of needed treatment (e.g., “[The side effects are] worth it if you get rid of something. It’s like going in for chemotherapy because if it kills off the cancer, who cares? You put up with the bad to get the good”).

#### Treatment efficacy

Patients referred to treatment efficacy by expressing hope that the treatment would have the desired benefit of curing the illness or getting rid of the virus from the body and restoring health: “I weighed whether I want to have hepatitis in me or at least try to get it out of my system. So to live a longer life, I decided that it was worth going through the treatment to try to change the course of the virus.” Some patients were aware that the existing treatments had a particular cure rate and expressed concern that the treatment may or may not be effective in their particular case: “I’m not going to take that kind of debilitating side effect for a 40 % cure rate. I’m not going to quit drinking for a 40 % cure rate. I’m not going to feel that sick for a 40 % cure rate.”

#### New treatment drugs in development

Patients expressed awareness that new medications were currently in development and would become available soon. This anticipation influenced decision-making because of their belief that the new treatment would be preferable to existing treatment. Most patients were optimistic about the promise of new medications, with many expressing that they would delay treatment in the hopes of “something better”: “Maybe something better will come down because that’s what everybody keeps telling me; every time I talk to the doctors, there’s something coming down the pike. We’ll be getting some new treatments come along and they won’t quite be so bad.” Similarly, another patient said, “I can’t remember what [my doctor] said the side effects and everything else was of the [current] treatment, but it didn’t sound like fun so I figured I’d wait for an easier treatment.” In contrast, a few patients felt that the wait would be too long when reasonable treatment was available now: “[My doctor] explained to me that they have a new treatment that it was in trials and it seemed promising for treatment of hepatitis C. She had said it seemed to look positive because the patients that were undergoing that seemed to be able to handle that treatment. But I was willing to undergo the treatment they have going now because I feel that I’m strong enough to undergo it.”

#### Psychological side effects

Patients expressed concern about the possible mental health side effects of interferon-based treatment as a consideration in their decision-making. They noted concerns about depression, anxiety, changes in moods, feeling irritable, worsening of post-traumatic stress disorder (PTSD) symptoms, or suicidal thinking. For example, one patient said, “The treatment that they’re offering now actually has a side effect that leaves you psychologically drained and mood swings and things like that. And so I was concerned that if I did that now with me being in school and employed, how would that affect me.” Some patients who had controlled depression or a history of depression worried about it becoming an issue again and this was a deterrent to treatment: “The treatments that are available now, they cause depression or whatever. And I’ve been kind of holding off on dealing with that because I’m already depressive and I didn’t want to exacerbate that. So that’s the main reason that I haven’t gone on with the treatment.” Other patients considered this attribute, but ultimately decided they were not concerned about mental health side effects impacting them if they chose treatment: “[Depression] was one of the [concerns], ‘cause I’ve never had depression. So, I didn’t know how to act on that. It said suicidal thoughts when I was reading the paperwork. I said, I don’t think I’m that bad off. I don’t think I’ll ever get that away. So, I decided to go on and try [treatment].”

#### Condition of the liver

Patients expressed concern about the health status of their liver including the stage of their liver disease, the presence or absence of cirrhosis, or other biomarkers such as viral count or liver enzymes. They tended to describe their liver health in general terms such as “bad/high/damaged” or “not so bad.” “Bad” liver health was seen as reason to treat the illness soon: “Well now that I know I’m at stage three, it’s a big concern on the liver. You know you only get one [liver], we’re just gonna try to get it fixed through what’s available for me right now.” “Not so bad” liver health was seen as a reason for possibly delaying treatment: “[My doctor] pretty much said that my enzymes are just I think one above the top [of the normal] range, so nothing’s really bad yet. I don’t know, so I haven’t really thought about treatment or whatever either. And she said I wouldn’t have to worry about it for a while basically.” Some patients were additionally concerned with the unpredictability of the progression of liver damage, and the potential irreversibility of reaching worse stages of liver health: “I just don’t know when it’s going to flip from stage three to stage four and that’s kind of worrisome. You know I was told stage four there’s no turning back; you know your liver’s gone when it goes to cirrhosis.”

### Analysis by patient group

We analyzed the frequency of decision attributes broken down by patient group. The number of attributes considered was similar across the groups. Group 1 patients (contemplating) on average reported slightly more attributes than Group 3 patients (recently started treatment), while Group 2 patients (recently declined treatment) reported the fewest attributes (8.75, 8.25, 7.75 attributes, respectively). All groups showed similar overall patterns with respect to the relative frequencies of the decision attributes they mentioned. However, Group 1 and 3 patients were more likely to identify treatment efficacy (92 % and 88 %) than Group 2 patients (56 %). Additionally, physical side effects were identified more frequently by Group 1 patients (92 %) than by Group 2 or 3 patients (78 % and 75 %). Fifty percent of Group 3 patients endorsed two decision attributesa (difficulty of frequent travel to VHA and urgency to treat) that did not appear in top attributes of the other two groups (Table 3).

**Table 3:** Decision attributes by patient group

	Group 1-Contemplating treatment	Group 2- Recently declined treatment	Group 3- Recently begun treatment
1	Efficacy/cure rate (92 %)	Physical side effects (78 %)	Efficacy/Cure rate (88 %)
2	Physical side effects (92 %)	Psych/Mental side effects (67 %)	Physical side effects (75 %)
3	New drug coming out (67 %)	Treatment regimen (67 %)	Other illnesses (63 %)
4	Quality of life (67 %)	Efficacy/cure rate (56 %)	Travel to VHA (50 %)
5	Psych/Mental side effects (58 %)	New drug coming out (56 %)	Urgency to treat (50 %)
6	Condition of liver (58 %)	Condition of liver (56 %)	

### Analysis by patient gender

We analyzed the frequency of decision attributes identified by male (*n* = 21) and female (*n* = 8) patients. Both male and female patients named physical side effects (88 % of women and 81 % of men) and treatment efficacy (88 % of women and 76 % of men) as important attributes in their decisions. However, female patients more frequently than males noted the impact of experiencing medical privacy and stigma concerns (50 % of women and 14 % of men) as decision attributes. Patients were concerned about maintaining privacy around their illness status among family members, coworkers, and community members, and the impact of social stigma associated with a communicable disease, either threatened or actually experienced. Female patients (25 %) also noted the impact of stress on primary partner relationships, and not having to pay for treatment in VHA, whereas none of the male patients mentioned these decision attributes. Thus, while women and men considered the same top decision attributes in their CHC treatment decision-making processes, social attributes such as stigma/privacy concerns and impact on primary partner relationships, as well as not having to pay for treatment in VHA, were more common among women.

### Relationships between decision attributes

**Fig. 1:**
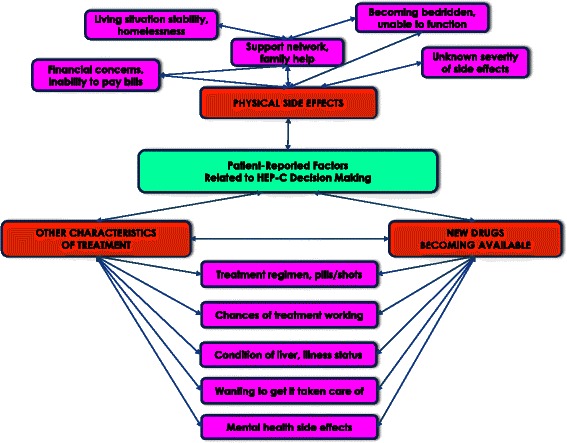
Selected Relationships Between Decision Attributes

Decision attributes were related to one another in highly complex ways within patient descriptions of their decision-making processes. Certain attributes frequently co-occurred, forming patterns in how patients tended to consider various attributes to conjunction with other attributes. Co-occurrence patterns were consistent across patient groups. Figure 1 illustrates selected relationships amongst decision attributes.

#### Physical side effects cluster

Physical side effects were closely related to a cluster of other decision attributes. The most common association was with family and friend support networks. Patients expressed concerns that the severity of physical side effects was unknown and it was difficult to predict if the side effects would prevent them from going about their usual routines. As a result, the availability of family and friend support networks becomes a related attribute of concern, should they require help. Closely related to this was the attribute of family financial obligations. For example, some patients were concerned that physical side effects may prevent them from working and paying bills: “I was wondering, am I going to be able to continue working [due to side effects while on treatment]? What’s it going to do with our finances? I mean there was just a lot of ‘what ifs’. What am I going to do?” This patient also noted concerns about efficacy of treatment, side effects, and finances: “Four years ago I said no [to treatment]. There is no way because doing the treatment was such a long process. Didn’t know how I was going to respond to the treatment. I did a lot of research on it and it didn’t sound like it was as effective as what I would think it should be. And plus the side effects, and I was worried about our finances of whether that I would be ill and not be able to go to work.” Some patients were concerned about the impact of side effects in light of the stability of their living situation, or on their ability to care for their families: “[My doctor] was unwilling to start me [on treatment] right now, because of the side effects. Plus my wife is disabled and I’m pretty much here with her. If something happened to me then there’s nobody here to take care of me.”

#### New drugs becoming available cluster

The promise of the on-going development of new CHC medications was also an attribute that frequently demonstrated co-occurrence with other decision attributes. When considering new treatments on the horizon, patients considered this attribute in conjunction with a constellation of other decision attributes including: characteristics of existing treatment, treatment regime, length of treatment, chances of treatment success, doctor’s recommendation, support networks, physical side effects, mental health side effects, liver status, progression of illness, social support, as well as a general sense of urgency or lack of urgency to address the issue of the illness in the patient’s life. For example, one patient explained, “I’m still not sure if I don’t want to take it or not. [My doctor] told me it’s better for me not to really take it at the stage I’m in right now. So if it’s come to a point where…I have to take it, yeah, I’m going to take it. But as of right now, he told me don’t. At the stage I’m in, don’t worry about it right now— they’re working on two different types [of new treatment] right now. I think the second one they’re coming up with, it don’t have no side effects at all or little side effects. So that’s the one he may introduce me to in the future.” Another patient noted the role of support in deciding to proceed with currently available treatment: “[My doctor] said there were some [future treatments] that would not have as severe side effects but I just went ahead and went on with the Pegasys. It’d be real nice if you can get cured and no drastic side effects. [But] I had the support to go ahead and do it [the currently available treatment] and it’s just a peace of mind for me. That it’s getting taken of care of; I’m not sitting here harboring something that could just get worse.”

## Discussion

Patients weigh a number of decision attributes when considering CHC treatment options. The decision attributes most frequently reported by patients were: physical side effects of antiviral treatment, treatment efficacy, new antiviral drugs in development, psychological side effects of antiviral treatment, and the condition of the liver. The number of attributes considered and the most frequently reported attributes varied somewhat based on the patient group (contemplating treatment, recently declined treatment, or recently begun treatment). Decision attributes were interrelated in patients’ decision-making narratives and this was consistent across groups. Relationships between decision attributes included: physical side effects and their unpredictable severity were frequently considered in conjunction with support networks and financial obligations, and the promise of new treatment drugs was considered in conjunction with a number of other attributes.

The overall patterns of most frequently mentioned decision attributes were similar across the three patient groups, with a few notable differences. The patients who were actively contemplating treatment considered slightly more decision attributes and were more likely to identify physical side effects than the other patient groups. These undecided patients may have been actively seeking as much information as possible to guide their decision-making. Patients who had recently started interferon-based treatment may have been motivated by an urgency to treat despite the promise of new drugs becoming available; these patients also noted the challenge of travel to VHA due to the frequent trips they made to receive treatment.

Our analysis by gender suggests that men and women typically consider the same decision attributes, but women may be more likely than men to consider certain social attributes (such as stigma and primary partner relationships) and financial attributes (such as the cost of treatment). Further study and larger sample sizes are needed to better understand the potential role of gendered decision attributes in patient decision-making around CHC treatment. These findings by groups and gender bring to light two important but often overlooked sets of decision attributes related to decision-making [38]. The first are logistical attributes (like travel and cost) that may moderate the relationship between behavioral intention and action. The second are affective states of urgency, or lack of urgency, that impact patients’ perceptions of timeliness, the desire to make and act on a decision within a certain timeframe, and peace of mind.

The most frequent decision attributes identified across the groups and between genders primarily reflected patients’ perceptions of medical states (liver health) and predictions of impact of treatment. However, other common attributes were tied to patients’ specific life circumstances and preferences. Patterns in the co-occurrence of decision attributes highlight the ways in which “medical” attributes and “life circumstance” attributes were closely woven together in patient experiences. Thus while patients’ treatment decisions were influenced by their personalized medical risks associated with their illness status, they were also influenced by the ways in which these risks interfaced with their unique life circumstances.

The need to consider personalized medical risk in conjunction with personal life circumstances and preferences [39], and the need to foster mutual engagement in care that is likely conducive to subsequent adherence and completion of complex treatment regimens [40, 41] makes shared clinical decision-making particularly germane to CHC treatment decisions. These results shed light on decision attributes that influence patient CHC treatment decisions and reveal complex relationships between medical attributes and life circumstance attributes in these decisions. They help illuminate common perceptions patients bring to the table in a shared decision-making context, and can be used to help inform a relevant decision-making aid.

Shared decision-making requires values clarification by patients involved in the decision-making process; quality standards exist for validation of decision aids, and these standards typically include a standardized process for values clarification [26, 42, 43]. Patients would likely benefit from a tool to help identify relevant decision attributes and their relationships, and clarify relative importance/impact on overall circumstances in order to make a decision that is not only consistent with their values, but also consistent with their personalized risk association with CHC [42]. Such a tool will need to take into account the attributes and relationships between attributes discussed here. In addition, findings by patient group and gender can help tailor versions of a decision-making aid for particular patient sub-groups.

While some qualitative studies have examined non-Veteran patient attitudes towards CHC treatment in general [17, 27–29] and others have examined treatment decision-making among individuals co-infected with HIV and CHC [30–32], to the best of our knowledge, this is the first qualitative study of Veterans’ rationales and thought processes regarding the initiation of CHC treatment. Although most of the top decision attributes endorsed by patients may not remain very relevant with the new interferon-sparing treatments, many attributes will still be important – such as condition of liver, other illnesses, life span, and the promise of newer drugs in development. Thus our work and the resulting tool will continue to have implications for antiviral treatment-related shared decision-making and can be modified or refined as new treatments become available.

These findings should be considered in light of several limitations. Our study was limited to four sites only. However, the sites represented small and large facilities and geographic diversity. The VHA serves primarily men; to account for this we purposely oversampled women for the study. This study was carried out entirely within the VHA healthcare system and as such, the findings may be particular to VHA users with CHC. Compared to non-Veteran populations, Veterans receiving care through VHA have a greater likelihood of psychiatric comorbidities and substance use, are more likely to end treatment early, and tend to respond to treatment at lower rates [44]. Finally, our qualitative methods are unable to determine the nature or strength of the associations among the decision attributes that we identify; future studies should examine this issue.

As the treatment landscape for CHC rapidly continues to change, more studies are required to understand the multidimensional and interrelated attributes that patients consider in their treatment decisions. The widening availability of new drugs with fewer side effects calls for additional studies to update our knowledge of how patients respond to new and different treatment choices. Because of the prevalence of individual life circumstance-related attributes, future work in this area should include a broad diversity of patients to increase our understanding of how distinctions such as race, socioeconomic class, education, substance use histories, and incarceration influence lived context for CHC decision-making. Due to the intensive nature in which patients undergoing CHC treatment interact with their healthcare system, further study is also warranted with Veterans who use other healthcare systems, as well as non-Veterans.

## Conclusions

Patients and providers face complex CHC treatment decisions within a dynamic context of changing options. Patients consider multiple decision attributes related to personalized medical risk as well as life circumstance, and these attributes are interrelated in complex ways. In the face of rapidly changing treatment options as new drugs become available, understanding the role of decision attributes that patients bring to the decision-making process is crucial. The prevalence of CHC in the general population, combined with the constantly evolving array of treatment options, make CHC decision-making a persistent issue with changing options for years to come both within and outside the VHA. As new treatments become available, these findings can guide clinicians to provide patient-centered decisional support to address key attributes that influence patients, and contribute to shared decision-making aids.
